# Influence of Hyperthermia Treatment on Varroa Infestation, Viral Infections, and Honey Bee Health in Beehives

**DOI:** 10.3390/insects16020168

**Published:** 2025-02-05

**Authors:** Xinjian Xu, Shujing Zhou, Jinrong Huang, Fa Geng, Xiangjie Zhu, Hossam F. Abou-Shaara

**Affiliations:** 1 College of Bee Science and Biomedicine, Fujian Agriculture and Forestry University, Fuzhou 350002, China; xuxinjian1982@126.com (X.X.); sjzhou118@126.com (S.Z.); 5220609008@fafu.edu.cn (J.H.); 3225602069@stu.fafu.edu.cn (F.G.); 2 Department of Plant Protection, Faculty of Agriculture, Damanhour University, Damanhour 22516, Egypt

**Keywords:** honey bee health, *Varroa* mite, bee viruses, hyperthermia treatment

## Abstract

The mite *Varroa destructor* is widely acknowledged as the most destructive threat to honey bee (*Apis mellifera*) colonies on a global scale. Varroa mite infestations in bee colonies are intricately linked with viral infections, collaboratively leading to diminished bee populations and accelerated colony losses. Extensive research has firmly established the correlation between varroa mites and viruses, underscoring the mite’s efficiency in spreading viruses among bees and colonies. The effective control of varroa mites is expected to result in a decrease in viral infections within bee colonies. Research suggests that thermal treatments (hyperthermia) present a viable approach to combat varroa mites, with studies demonstrating the role of heat stress in reducing viral infections in affected bees. This article examines the extant literature surrounding the utilization of hyperthermia as a potential method to ameliorate the adverse impacts of varroa mites and their associated viral infections on honey bee colonies. It also outlines the thermal characteristics of these stressors. Diverse devices can be used for subjecting colonies to hyperthermia treatment, targeting mites both within and outside of brood cells. The application of thermal treatments, typically ranging between 40 and 42 °C for 1.5–3 h, as a method to reduce varroa mites and viral infections, has shown promise. Notably, the precise effectiveness of hyperthermia treatment in comparison with alternative varroa mite control measures remains uncertain within the available literature. The potential deleterious repercussions of this control mechanism on immature and mature honey bees are evaluated. Concurrently, the detrimental implications of prolonged treatment durations on colonies are discussed. Regarding viral infections, hyperthermia treatment can impact them negatively by either reducing varroa mite infestations or by inducing the production of heat shock proteins that possess potential antiviral properties. Various factors are identified as influential on hyperthermia treatment efficacy within bee colonies, including the device type and treatment duration, necessitating further empirical investigations. Additionally, this article highlights the existing gaps in the knowledge and provides insights into the prospective directions of research concerning this control method.

## Simple Summary

Research has suggested the utilization of thermal interventions, specifically hyperthermia, as a viable method for combating Varroa destructor mites. Simultaneously, studies have elucidated the impact of heat stress in reducing viral infections among afflicted bees. This article aims to conduct a review of the current literature on hyperthermia, providing an overview and discussion of the potential implementation of hyperthermia to mitigate the negative effects of varroa mites and associated viral infections on honey bee colonies, while outlining the thermal characteristics of these stressors. The article advocates for further research into hyperthermia treatment to address the challenges posed by varroa mites and viruses.

## 1. Introduction

Honey bees, *Apis mellifera*, are primary plant pollinators, and their substantial contribution to the global economy is significant [1,2,3], notably contributing to global crop production [4,5]. Beyond plant pollination, the importance of honey bees extends to enhancing the quality and quantity of crops [6] and supplying the market with a variety of valuable bee products [7,8,9]. Beekeeping, as an important economic activity, has garnered significant attention over the years, as evidenced by the rise in the number of managed colonies worldwide from 1961 to 2017 [10]. On the other hand, the rate of honey bee colony losses under specific conditions has also increased, presenting a significant challenge to agricultural development and food production in affected regions. This loss has been documented in several countries, with potential negative effects on the pollination of plants by honey bees [11,12]. Various biotic and abiotic factors can contribute to this loss. Among them, *Varroa destructor* mites (Anderson and Trueman 2000) and viruses hold special importance, due to their global distribution [13,14,15].

Varroa mites have been detected worldwide [16,17], including in Australia, which has long been recognized as varroa-free [18]. This global distribution underscores their status as a major challenge for honey bee colonies. Their ability to climb from flowers onto foraging honey bees [19] and their potential spread via natural bee swarms and commercial bee packages [20,21] underscore their multiple strategies for infecting honey bees. The damages associated with varroa infestations include stunted colony development and reduced productivity [22,23]. At the individual level, infected bees experience compromised immunity and health, along with decreased reproductive capabilities in male bees [24,25]. Moreover, varroa mites play a significant role in the transmission of viruses among individuals, colonies, and apiaries [26,27,28], amplifying their indirect harmful effects on honey bees.

Regarding viruses, a multitude of viruses have been identified in honey bee colonies [29]. Some of these viruses can inflict severe damage on developmental-stage bees, such as larvae and pupae, including sacbrood virus and black queen cell virus [30,31]. Additionally, deformed wing virus exhibits symptoms in emerged adults as well [13,32,33]. Other viruses can cause significant harm to adult bees, like paralysis viruses such as chronic bee paralysis virus [34] and acute bee paralysis virus [35]. It is common for honey bee colonies to harbor a combination of viruses simultaneously [36,37]. These viruses substantially contribute to impeding colony development and compromising survival rates [38,39]. Various modes exist for the transmission of these viruses from one bee to another and from one colony to another [40]. Colonies infested with varroa mites are closely associated with virus infections in bees [41,42]. The concurrent presence of these two biotic stressors within a colony can substantially diminish its chances of survival.

Given the widespread distribution of varroa mites and significant harm inflicted by them on bee colonies, a variety of methods have been suggested to combat them [43]. These control strategies encompass a spectrum of chemical and non-chemical approaches. Formic acid is utilized to combat varroa mites inside and outside brood cells, while varroa mites outside brood cells can be managed using essential oils, oxalic acid, acaricides, and by dusting bees with powdered sugar or flour [8,44,45,46]. Furthermore, an effective method for mitigating the negative effects of varroa mites on honey bees involves employing thermal treatments, such as hyperthermia devices [47,48]. Heat stress exposure has also been suggested as a way to detrimentally affect viruses [39,49,50]. This review aims to provide an analysis of the existing knowledge regarding the attributes of hyperthermia treatment used to combat the varroa mite and its associated viruses, as well as the tolerance and impact of this type of treatment in the honey bee colony.

## 2. The Association Between Varroa Mites and Viruses in Colonies

Varroa mites and viruses can be detected within infected colonies [41,42,51,52]. While viruses can be found in mites, they can also be identified in colonies free of mites [53,54]. The incidence of mites in bee colonies is intricately linked to colony growth, a process that has a seasonal pattern [55,56]. The heightened brood-rearing activity observed during specific seasons, such as spring and summer in regions with a temperate climate, acts as a stimulus for mite reproduction. This phenomenon is elucidated by the significant role of brood cells in the reproductive cycle of varroa mites, resulting in the production of 0.7–1.45 and 1.6–2.5 mature female daughters in worker brood cells and drone cells, respectively [17,25]. Juvenile mites hatch from eggs within these cells, mature, and then undergo mating [17,25]. Conversely, phoretic mites tend to be more prevalent in bee colonies during the autumn and winter periods when brood production wanes [57]. Since varroa mites act as conduits for viral dissemination within colonies, viruses generally exhibit more autonomy in their presence compared to the seasonal patterns of mites within bee colonies (e.g., 39). This autonomy can be attributed to the existence of multiple pathways for viral transmission among bees and colonies. Some viruses, such as deformed wing virus, can be vertically transmitted during egg laying and horizontally transmitted through interactions between infected and healthy bees [58,59,60]. Furthermore, the transmission of viruses such as deformed wing virus, Israeli acute paralysis virus, and black queen cell virus among pollinators is feasible [61,62,63,64]. Despite transmission routes not involving varroa mites, under conditions of co-infection, colony damage is exacerbated.

## 3. Effects of Temperature on Varroa Mites and Viruses

The life cycle of varroa mites pertains to the ability of the immature-stage insects to complete their development within the brood cells of honey bee colonies [65]. This implies that temperatures of 32–36 °C in and around the brood area are not harmful to varroa mites both inside and outside the cells, unlike temperatures of 40 °C or higher, which can be lethal to them [66]. Immature-stage honey bees can endure temperatures of up to 43 °C for short periods (<8 h), while adults can withstand temperatures of up to 48 °C without significant harm [67,68,69,70]. Conversely, reduced reproduction of female mites can occur at temperatures of above 36.5 °C, while temperatures exceeding 38 °C can induce mortality in mites without reproduction [71]. The exposure of mites in brood cells to a high temperature of 44 °C for 4 h shortly before the emergence of adult bees resulted in 100% mortality [72]. When the mites latch onto adult forager bees, they may encounter extreme temperatures outside the hives for brief periods. This corresponds with the few seconds or minutes that foragers typically spend at each feeding site or flower [6,73,74].

Varroa mites can survive away from their host, but only for a limited duration. Studies on the longevity and survival of varroa mites under diverse conditions are still limited, emphasizing the necessity for additional research in the future. In a laboratory study where mites were exposed to 29 °C, 40 °C, or 50 °C for 30 min, phoretic mites exhibited 100% mortality at 40 °C. However, all mites collected from inside the cells of brood combs were alive at this temperature. Conversely, all mites were alive at 29 °C, but all of them died at 50 °C [75]. Such variations necessitate additional investigations to figure out the differences between varroa mites at different stages in their ability to withstand exposure to high temperatures. The role of heat shock proteins (HSPs) in protecting cells from damage during heat stress is well documented in honey bees and various other organisms [76,77,78,79,80]. Hsp70 can be significantly induced in both phoretic mites and those from brood cells in response to heat stress and acaricides [75]. Despite the potential antiviral role of HSPs [80], their impact on viral loads in varroa mites remains undocumented.

Weather conditions, particularly temperature, have shown potential impacts on the prevalence of viruses in bee colonies [39,49,81]. Certain viruses demonstrate increased prevalence during periods of low temperature, such as winter, for example, the sacbrood virus in Southwest Germany [82], while others are more prevalent in high-temperature periods like summer, such as the black queen cell virus in France [83] and Greece [84]. Optimal brood temperatures (33–36 °C) are conducive to the survival and replication of certain viruses in bee colonies, such as the black queen cell virus [85] and deformed wing virus [86]. Extreme temperatures are anticipated to influence viral loads in infected bees. Moreover, honey bees aged 24 h post emergence and maintained at 32 °C were injected with either buffer or virus. Subjecting the bees to a heat shock of 42 °C for 4 h triggered a heat shock response in honey bees, and heat-shocked bees showed a 74–90% lower mean virus abundance compared to bees maintained at 32 °C [50]. This highlights the potential advantage of exposing infected bees to heat stress as a strategy to alleviate the detrimental effects of viruses on honey bee populations.

## 4. Hyperthermia Treatment

The principle of using hyperthermia to control the varroa mite population relies on the differing thermal tolerances of various developmental stages of honey bees, including brood and adult individuals, as well as the developmental stages of varroa mites and their adult females (foundress and phoretic), in relation to exposure time. A temperature of 40 °C has been identified as the minimum threshold required to eliminate mites from infected caged bees over a 48 h period [87]. Various devices have been developed for varroa mite control using hyperthermia. These include the mite zapper in the USA, which targets reproductive mites in drone brood [88]; the thermovar in Greece, which impacts reproductive and phoretic mites [89]; the thermo-solar hive [47]; the Bee Ethic system in Italy, comprising an electronic control unit and a set of heated frames [48]; and the vatorex in Switzerland, which targets reproductive mites in brood cells [90]. These devices expose mites to high temperatures ranging from 40 to 42 °C for 90–180 min, with the aim of adversely affecting mites.

Hyperthermia treatment should carefully adhere to an exposure time of approximately 1.5–3 h. Prolonged exposure to 42 °C for 4–8 h can result in increased brood mortality [89], as detailed in the next chapter. A well-implemented heating treatment can reduce infestation rates of varroa mites and potentially mitigate the associated viruses. Supporting this, the use of hyperthermia has demonstrated negative impacts on the viral loads of deformed wing virus (DWV) and acute bee paralysis virus (ABPV) in worker samples from treated colonies, compared to untreated ones using the Bee Ethic system [48]. This may be attributed to the detrimental effects of hyperthermia on varroa mites and their associated viruses, or the direct impact of heat on viruses. HSPs play a crucial role in influencing virus replication [91,92,93]. Therefore, subjecting varroa mites to heat stress can trigger the expression of HSPs in their bodies [75], potentially affecting viral loads in surviving mites post heat treatment. However, there are currently no available data on whether viral replication is activated or inhibited in varroa mites exposed to heat stress for a specific duration, warranting further in-depth studies. Similarly, the impact of HSPs on reducing viral loads in infected bees has not been thoroughly investigated following the use of hyperthermia for varroa mite control. It appears that many aspects highlighted here necessitate further investigation, and are currently lacking in the existing literature.

The potential effects of hyperthermia on varroa mites inside brood cells when heating the whole hive are debatable; particularly, honey bee workers may shield the broods to protect them from overheating [70]. There are devices that target reproductive mites inside brood cells, such as the mite zapper, a drone comb embedded with heating elements to control varroa mites inside infested drone brood cells [88], but it has not been widely used on a commercial scale. A study showed that a thermal treatment of 41 °C for 2 h has no negative effects on drones, while it causes harmful effects on immature-stage varroa mites inside brood cells [94]. The heat treatments employed to control varroa mites inside sealed brood cells are expected to have minimal effects on honey bee broods; in particular, larvae have a higher ability than mites to tolerate heat, by 2–3 °C [67]. Devices that target heating brood combs only for about 2 h, instead of the whole hive, are more time-consuming and less practical.

In terms of efficacy, the current studies are insufficient to provide a comprehensive understanding of the effectiveness of hyperthermia treatments against varroa mites under various conditions. Factors such as the type of device used, application timing, and colony strength collectively influence the efficacy of hyperthermia treatments. For instance, when comparing control and hyperthermia hives in Italy utilizing the Bee Ethic system—raising the brood temperature to 42 °C for 90 min every three weeks from May to mid-October—the mortality rates of juvenile varroa mites in hyperthermia hives ranged from 79.9% to 93%, and for adult varroa mites from 24.8% to 43.5%, showing lower infestations compared to the control group [48]. In another experiment conducted in Greece utilizing the thermovar, known to impact reproductive and phoretic mites, colonies underwent treatment at 42 °C for durations ranging from 12 min to 8 h. This led to a notable escalation in internal hive temperatures, elevating them to 42.3–46.5 °C. Following a 24 h period post treatment, the mortality rates of varroa mites on the hive’s bottom board surged from 4.6% (with a treatment duration of 30 min) to 45.5% (with a treatment duration of 8 h). Furthermore, mite mortality within brood cells surged from 4.7% (with a treatment duration of 60 min) to 100% (with a treatment duration of 8 h), underscoring the critical role of treatment duration in the effectiveness of hyperthermia [89]. It is evident that hyperthermia treatment can induce mortality in varroa mites both inside and outside brood cells. However, further studies should be conducted to precisely determine the efficacy of this treatment method under varying conditions. The existing comparisons between hyperthermia treatment and other varroa mite control methods are insufficient to determine the extent to which hyperthermia may surpass the alternative approaches. Therefore, conducting comparative studies on hyperthermia treatments utilizing various devices to target varroa mites within and outside of brood cells, in conjunction with other varroa mite control methods, is imperative for addressing the current knowledge gaps.

## 5. Effects of Hyperthermia on Honey Bee Colonies

On an individual level, honey bees are ectothermic insects that lack the ability to maintain a constant body temperature. On a colony level, honey bees regulate brood temperature within a range of 33 to 36 °C [95,96,97], which is crucial for colony development and survival [70,98,99]. Honey bees employ several mechanisms to adjust colony temperature during periods of heat stress, such as evaporative cooling, fanning behavior, and heat shielding mechanisms [70,100,101,102]. The exposure of adult bees shortly before emergence to 44 °C for 4 h did not cause significant negative effects on emergence rate or lifespan, unlike exposure to a temperature of 45 °C [72]. Short-term exposure of bee colonies to heat stress (40–42 °C), as applied during hyperthermia treatment of colonies, is generally not expected to have harmful effects on the adult bees. An observable change may be the increased levels of HSPs during this exposure period [80], necessitating further in-depth studies. Under normal colony conditions, the induction of certain HSPs serves as an adaptive resistance mechanism against colony overheating [103]. Under laboratory conditions, the survival rates of bees collected directly from colonies (~35 °C) and exposed to heat stress (45–47 °C) were higher compared to those acclimated to 20 °C [103].

In terms of behavior, bees are anticipated to actively counteract the impacts of heat stress. This may involve actions such as congregating outside the colonies to lessen the heat generated within, enhancing fanning behavior, and temporarily adjusting foraging patterns to gather water for evaporative cooling purposes [70,100,101,102]. When considering the broods, short exposures to heat stress (40–42 °C) may have varying effects based on the developmental stage. Eggs may exhibit decreased hatching rates as a result of temperature and relative humidity acting as limiting factors for egg viability, which should be optimal for the brood area [68]. Conversely, elevated temperatures reaching 40–42 °C could result in increased mortality during the egg and early larval stages. The use of hyperthermia through heating brood combs has demonstrated effectiveness in reducing varroa mite populations until autumn [90]. This method results in increased egg and larval mortality, subsequently reducing the population of adult workers and honey production [90]. Pupae are generally expected to complete their development normally, but there may be subsequent effects on the behaviors of emerged workers, necessitating further research. Research has shown that exposing broods to a range of temperatures from 29 °C to 37 °C during development can lead to behavioral and physiological abnormalities, such as abnormal waggle dance performance, compromised learning, and impaired memory [97,104,105]. However, brief exposures to heat stress are expected to have minimal effects on both broods and emerged adults. For instance, short-term hyperthermia exposure during the larval stage (at 43.7 °C for 2 h) resulted in reduced sucrose responsiveness in adult bees, but extended their lifespans [106]. This suggests that hyperthermia for varroa mite control does not significantly impact the survival of larvae or emerged adults, although it may lead to slight behavioral changes. Further studies are needed to explore this aspect in more detail.

Queens are expected to cease egg laying during periods of stress, often with some workers gathering around them [46,70]. However, the death of queens is not anticipated. Studies have indicated the potential negative impact of heat stress on queens’ fertility [107], including negative effects on the viability of stored sperm in the spermatheca following thermal stress of ≥40 °C for approximately 2 h [108]. Contrary to this, exposure to 42 °C for 2 h, a temperature similar to that used in varroa mite treatment, has shown no detrimental effects on stored sperm viability in queens [109], suggesting the resilience of queens’ reproductive performance following stress exposure. Given that a single queen typically dominates the colony under normal circumstances, it is advisable to temporarily remove queens from colonies during treatment periods. This can be achieved by placing the queen in a cage and relocating it to new empty boxes until the treatment concludes, or by confining the queen on a comb and removing it during the treatment. This suggestion aims to guarantee the well-being and performance of queens post hyperthermia treatment. However, conducting detailed studies on this matter is crucial to provide a clear understanding of queen health and determine the necessity of implementing specific protective measures for queens.

## 6. Outlooks

The potential of utilizing thermal treatment (hyperthermia) for controlling varroa mites has been largely underexplored in the existing literature, leading to a lack of comprehensive comparative analyses between thermal treatments and other chemical and non-chemical methods for varroa mite control. The extended time required to treat a single colony, typically spanning from 1.5 to 3 h, and the necessity of employing a dedicated device for each hive to individually manage varroa infestations across multiple bee colonies simultaneously, may have hindered commercial beekeepers from considering this method compared to alternative control options. Currently, the application of hyperthermia for varroa mite control is mostly limited to hobbyists and non-commercial beekeepers who maintain only a small number of colonies.

Further research is essential to enhance our comprehension of the actual impacts of thermal treatment on varroa mites, viruses, and bees. The ideal timing and frequency of treatment remain topics of ongoing discussion. Recommendations advise against exposing broods during crucial developmental stages when employing hyperthermia for varroa mite control [106]. Optimal timing for thermal treatment, focusing on targeting phoretic varroa mites while minimizing the effects on broods, may align with periods like autumn, when brood-rearing activity notably declines. Additional practices such as queen caging or ringing for brood interruption in colonies [110,111] can be implemented before treatment to ensure the absence of unsealed broods during the treatment period. To mitigate the potential negative effects on broods while using Vatorex AG (Venturelab Ltd., Schlieren-Zurich, Switzerland), which applies hyperthermia to brood combs, it has been suggested to reduce the treatment temperature and exposure time. For example, using 41 °C for 70–100 min instead of 42.5 °C for 130 min, as this adjustment can primarily affect mother varroa mites and not immature varroa [90]. However, further studies are needed to investigate the advantages and disadvantages of reducing the treatment temperature and exposure time for hyperthermia devices targeting mites in brood cells. Given the morphological diversity and haplotypes of varroa mite populations [65,112], it is vital to investigate how thermal treatment impacts them, especially considering that mites with varying morphologies have shown differences in pesticide tolerance [113]. Comprehensive studies are necessary to identify the most effective intervals, temperatures, and durations for repeated thermal treatments to minimize varroa mite and virus infestations. Incorporating hyperthermia into integrated pest management (IPM) strategies for varroa mite control has been proposed [48], since hyperthermia is recommended during periods without high brood-rearing activity. As a suggestion, hyperthermia alone can be effectively used with bee packages (boxes containing adult bees without broods), particularly prior to introducing caged queens into the packages. By placing bee packages in rooms that have thermal control options, treating hundreds of bee packages can be achieved cost-effectively and swiftly. Further intensive research is warranted for this dual treatment approach targeting varroa mites and viruses to establish practical guidelines.

When considering honey bee colonies, it is essential to take into account the subspecies of honey bees. Different honey bee subspecies vary in their ability to withstand heat stress. For instance, Yemeni honey bees, *Apis mellifera jemenitica*, demonstrate a higher tolerance to high temperatures compared to Carniolan, *Apis mellifera carnica*, or Italian bees, *Apis mellifera ligustica*, and their hybrids [114,115]. Therefore, when employing thermal treatments to control varroa mites, it is crucial to test these treatments with each subspecies individually, rather than applying results blindly from one subspecies to another. This becomes particularly crucial if honey bee queens are not intended to be removed from colonies before treatment, as queen failure can be associated with exposure to heat stress in certain scenarios. Additionally, the heat tolerance of queens from different bee subspecies remains a debatable point. Furthermore, the material of the beehive, whether plastic or wood, can impact the thermoregulation of honey bees within the nest [70,116]. Therefore, the effectiveness of hyperthermia devices may be influenced by the type of beehive, highlighting the need for thorough further studies in this area.

## 7. Conclusions

This article reviews the various facets directly or indirectly associated with the application of hyperthermia treatment (40–42 °C for 1.5–3 h) in the control of varroa mites within bee colonies, while underscoring its potential efficacy in mitigating viral infections. Despite its potential as a dual solution for addressing varroa mite infestations and viral threats, this approach has been notably understudied in previous scholarly investigations, resulting in significant knowledge gaps. Currently, the effectiveness of hyperthermia treatment in comparison with other available control measures for varroa mites remains inadequately researched. Various hyperthermia devices are currently accessible for combating varroa mites within and outside of brood cells; however, the potential harm to honey bees necessitates further investigation. Immature-stage bees can be negatively affected by hyperthermia, especially under prolonged exposure conditions. This underscores the recommendation that periods characterized by low brood populations within colonies may be optimal for implementing this control method. In terms of its negative impact on viral infections, the reduction in varroa mites or the induction of heat shock proteins in treated bees and varroa mites may help elucidate its potential effects. From a commercial perspective, the implementation of this treatment approach presents logistical challenges when addressing a large number of colonies. However, given its purported safety for honey bees and its chemical-free nature, which avoids contamination of honey bee colonies, there is a strong argument advocating for ongoing research and innovation in this field. With the persistent challenges posed by varroa mites and viruses, it is anticipated that this control strategy could play a crucial role in shaping future approaches to managing these stressors.

## Data Availability

This is a review manuscript, and no data are available from it.
